# International graduates’ experiences of reflection in postgraduate training: a cross-sectional survey

**DOI:** 10.3399/BJGPO.2021.0224

**Published:** 2022-05-04

**Authors:** Laura Emery, Ben Jackson, Phillip Oliver, Caroline Mitchell

**Affiliations:** 1 Academic Unit of Primary Care, The University of Sheffield, Sheffield, UK

**Keywords:** Reflection, postgraduate education, international graduates, general practice, primary healthcare, medical schools

## Abstract

**Background:**

Reflection is a key component of postgraduate training in general practice. International medical graduates (IMG) are thought to be less familiar with reflection, with international medical schools favouring more didactic methods of education.

**Aim:**

To explore IMGs’ experiences of reflection prior to and during GP training and the support available for developing skills in reflection.

**Design & setting:**

A cross sectional survey was sent to IMGs undertaking GP training in 12 of the 14 UK regions, from March to April 2021.

**Method:**

A pre-tested self-administered online questionnaire was used to collect data on experiences of reflection, both prior to and during GP training, and the support available for developing skills in reflection.

**Results:**

In total, 485 of 3413 IMG trainees completed the questionnaire (14.2% response rate, representative of national demographics). Of these, 79.8% of participants reported no experience of reflection as an undergraduate and 36.9% reported no formal training in reflection during GP training. The majority (69.7%) of participants agreed that reflection was beneficial for their training and 58.3% reported that the best support in reflection came from their supervisors. Experience of reflection, opinions on the benefits, and best sources of support all varied by where the responders' primary medical qualification (PMQ) was obtained (all *P* values<0.01).

**Conclusion:**

Most IMGs have not experienced reflection prior to commencing UK GP training. There is diversity in experience and culture within this group that must be considered when tailoring educational interventions to support IMGs in their transition to UK GP training.

## How this fits in

This study confirms previous assumptions that international graduates are unlikely to have experience of reflection before entering GP training. Support in developing skills in reflection is very variable depending on region in which GP training is undertaken. There is wide diversity in experience of reflection, views on benefits, and preferred support within this group of trainees. Educational interventions developed to support IMGs should be tailored to reflect the diversity within this group.

## Introduction

Differential attainment between IMGs and their UK trained colleagues remains a major problem in postgraduate education, both during training^1^ and in postgraduate exams.^2^ The evidence for educational interventions to support international graduates during training is weak, the research is often lacking in scientific rigour, and lacks an appreciation for differences in cultural background or previous experience.^3^

Reflection is applied across all disciplines in postgraduate medical education as a means of personal and professional development, which can identify opportunities for improvements in patient care.^4^ Reflection is assumed to be a new concept for many international graduates, with most describing their previous education as ‘science-orientated’.^5^ IMGs have been shown to favour didactic, teacher-focused educational techniques.^6^ This change in learning culture puts further pressure on international graduates as they adapt to living and working in the UK.^6^ This is likely to be amplified in GP training, where reflection is used to evidence General Medical Council (GMC) competencies for progression to Certificate of Completion of Training (CCT).^7^

It has been suggested that earlier introduction to and understanding of reflective practice could aid progression of IMGs in GP training.^8^ This is, however, based on assumptions about IMGs’ lack of familiarity and understanding of reflection, which has not been conclusively evidenced. The aim of this research is to explore IMGs’ experiences of reflection, both prior to and during GP training, including the support available for developing skills in reflective practice.

## Method

A cross sectional survey of all IMGs enrolled in UK GP training schemes was conducted from the 8 March to the 22 April 2021 using a pre-tested self-administered online questionnaire. At the time of the study, IMGs accounted for 37.5% of GP trainees in the UK, meaning 3627 trainees were eligible for inclusion in the survey.

### Study setting

Before applying for GP training in the UK, IMGs must first successfully complete the professional and linguistics assessment board test, which is an assessment of clinical knowledge and skills through written and structured clinical exams. To access a training programme, they must then provide evidence of foundation level medical competence and be successful in the national recruitment process.

There are 14 regional schools of primary care across the UK that oversee GP specialty training programmes. Applicants are required to choose their preferred programme and region when submitting their application. Appointment to a programme in a preferred region is highly dependent on competition ratios for that region, with generally more competition for London and the South East compared with other parts of the UK. Numbers and proportion of IMGs vary widely between region; in London 101 of 1131 trainees (8.9%) are IMGs compared to 556 of 979 trainees (56.8%) in the West Midlands. Within each region, competition also exists for the most popular training programmes and the proportion of IMGs can vary markedly between programmes within regions.

### Survey instrument

As there were no validated questionnaires available to evaluate this topic area, survey questions were designed by the lead researcher following a comprehensive literature review. Questions were supplemented by expert review from a panel of stakeholders and co-investigators, and content, phrasing, and layout were refined following piloting. Following an introduction that explained the nature of the study, including descriptions of members of the research team and funding body, there followed 5 sections of questions, listed below:

- Section 1: declaration of consent and demographic information
- Section 2: understanding of the purpose of reflection (open ended questions)
- Section 3: experiences of reflection prior to GP training (multiple choice questions)
- Section 4: training and support in developing reflection (multiple choice questions)
- Section 5: advantages and disadvantages of reflection (Likert scales and open ended questions)

To ensure all participants had some understanding of reflection, a definition of reflection and examples of the context in which it is used in postgraduate training were provided.

The survey was designed and applied using the Online Surveys tool. Participant information including a link to the online survey was sent via email to the heads of all the UK regional training schemes to be cascaded in a top-down approach. Two weeks before closure of the online survey, a further email was sent encouraging circulation of a reminder to trainees to maximise response rates.

An incentive for completion of the survey was offered in the form of entry into a prize draw for the chance to win an online shopping voucher.

### Data analysis

Data from closed ended questions were transferred into SPSS (version 27) software for analysis. Actual numbers and proportions were calculated for each question response. Pearson's χ2 tests were applied in order to make intergroup comparisons between the responses obtained from trainees of different sexes, age, previous medical experience, and subregion of the world in which PMQ was obtained. Comparisons between regional primary care schools were conducted.

Free text answers were imported into NVivo (version 1.5.1) software. Qualitative thematic analysis is ongoing and is not reported here.

### Stakeholder participation and involvement

Prior to commencing the research, a stakeholder group was recruited consisting of current GP trainees who achieved their PMQ outside of the UK and GP trainers with experience of training or studying abroad. This stakeholder group has been involved at all stages of the research, from design through to analysis and dissemination.

## Results

The questionnaire was sent to all 14 UK regional schools of primary care and was circulated by 12 of the 14 schools. The Wales and London schemes did not circulate the questionnaire to their trainees, despite several attempts to contact senior regional training figures. Excluding the 214 trainees who did not receive the questionnaire in these regions (113 in Wales and 101 in London), 485 of the 3413 remaining trainees completed the questionnaire (14.2% response rate).

### Regional response rates

**Figure 1.**
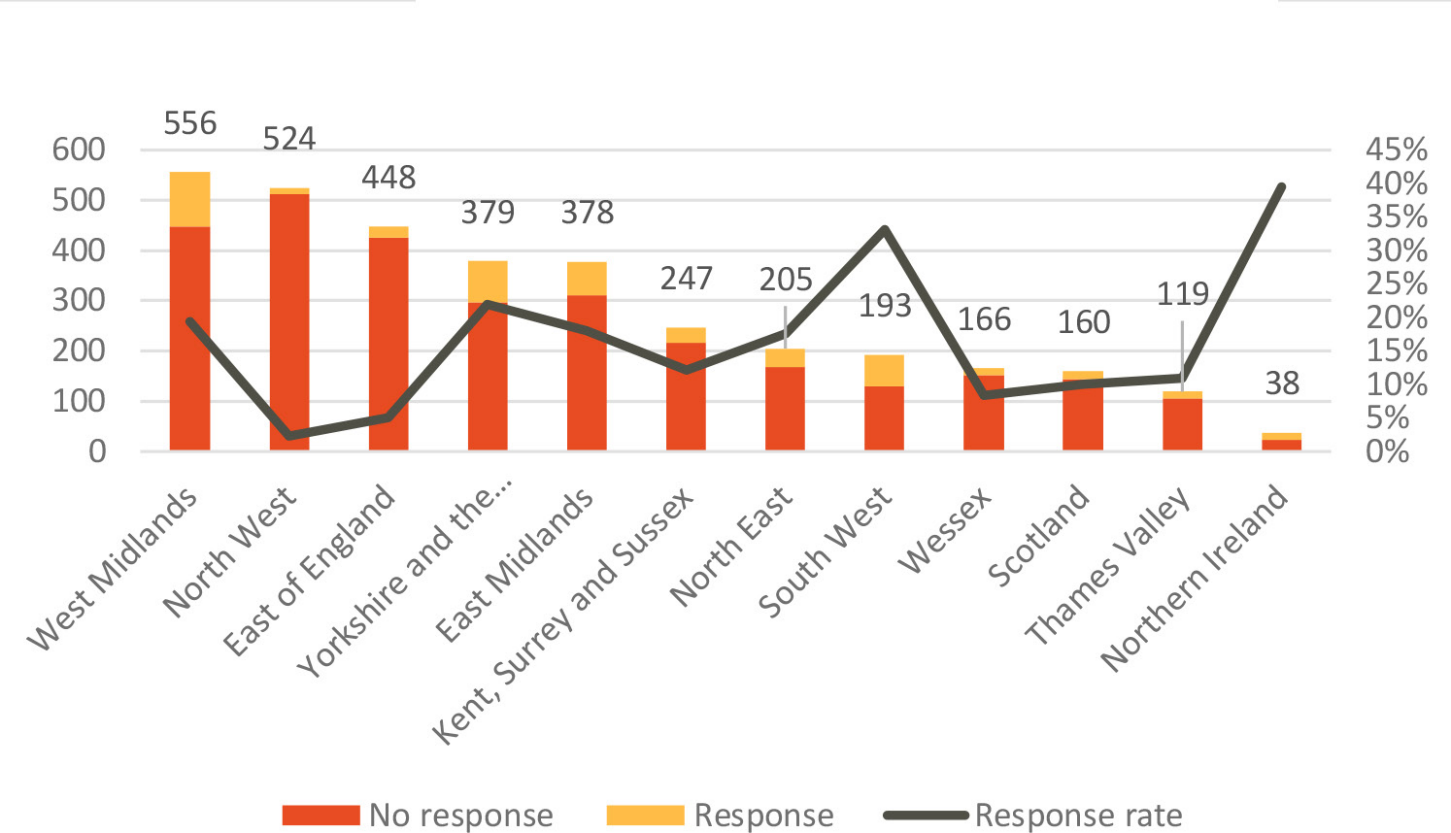
Response rates per UK regional primary care school.

Response rates varied greatly between region (Figure 1). The highest rates were in Northern Ireland (39.5%; 15 of 38 trainees) and South West England (33.2%; 64 of 193 trainees). The lowest rates were in North West England (2.3%; 12 of 524 trainees) and East of England (5.1%; 23 of 448 trainees)

### Demographics

The demographic characteristics of the responders are presented in Table 1 alongside the corresponding contemporaneous GMC data for GP training in the UK. The sample is broadly comparable to the GMC data in terms of sex, age, and subregion of the world in which PMQ was obtained. The country of PMQ was requested from all participants. To allow comparison with GMC data, these have been grouped together and presented according to subregions of the world. Most participants achieved their PMQ in Africa (35.7%; 173 of 485) and South Asia (32.2%; 156 of 485).

**Table 1.** Demographics of questionnaire participants comparisons with GMC data.

	Number of participants	Percentage of participants	NumberGMC data	PercentageGMC data
**Sex^a^**				
Female	259	53.4%	1911	56.0%
Male	222	45.8%	1502	44.0%
Total	481		3413	
**Age**				
25–29	44	9.1%	203	5.9%
30–34	195	40.4%	1164	34.1%
35–39	144	29.4%	1019	29.9%
40+	102	21.1%	1026	30.1%
Total	485		3412	
**Subregion PMQ**				
Africa	173	35.7%	1162	34.0%
Europe and Oceania	72	14.8%	521	15.3%
EAA Europe	59	12.2%	402	11.8%
Non-EAA Europe	11	2.3%	117	3.4%
Oceania	2	0.4%	2	0.1%
Middle East	49	10.1%	263	7.7%
Rest of the World	35	7.2%	207	6.1%
Rest of Asia	22	4.5%	130	3.8%
South/ Central/ Latin America and Caribbean	13	2.7%	77	2.3%
South Asia	156	32.2%	1260	36.9%
Total	485		3413	

EAA = European Economic Area. GMC = General Medical Council. PMQ = primary medical qualification.

^a^’Prefer not to say’, *n* = 4.

### Experience of reflection prior to GP training

When asked to rate their level of agreement with the statement ‘I experienced reflection as an undergraduate’, 20.2% of participants (98 of 485) selected ‘Agree’ (Table 2). Differences in agreement to this question were observed depending on subregion of the world in which the trainee's PMQ was obtained. Trainees who achieved their PMQ in Africa or the Middle East were significantly less likely to agree that reflection was part of their undergraduate training (Table 2).

**Table 2.** Survey responses crosstabs analysis with subregion of PMQ.

	Africa	Europe and Oceania	Middle East	Rest of the world	South Asia	Total	*P* value
**I experienced reflection as an undergraduate…**							
Agree	25 (14.5%)	17 (23.6%)	4 (8.2%)	13 (37.1%)	39 (25.0%)	98 (20.2%)	<0.001
Disagree	118 (68.2%)	51 (70.8%)	37 (75.5%)	18 (51.4%)	82 (52.6%)	306 (63.1%)	
Unsure	30 (17.3%)	4 (5.6%)	8 (16.3%)	4 (11.4%)	35 (22.4%)	81 (16.7%)	
Total	173	72	49	35	156	485	
**The best support and advice I have received in reflection is from…**							
I do not feel supported	15 (8.7%)	10 (13.9%)	8 (16.3%)	6 (17.1%)	16 (10.3%)	55 (11.4%)	0.007
Supervisors(CS/ES/TPD)	86 (49.7%)	45 (62.5%)	26 (53.1%)	19 (54.3%)	106 (68.4%)	282 (58.3%)	
Friends/peers	63 (36.4%)	14 (19.4%)	15 (30.6%)	7 (20.0%)	30 (19.4%)	129 (26.7%)	
Other (Self directed/online)	9 (5.2%)	3 (4.2%)	0	3 (8.6%)	3 (1.9%)	18 (3.7%)	
Total	173	72	49	35	155	484	
**Reflection as I have experienced it in UK GP training is beneficial…**							
Agree	141 (95.3%)	43 (74.1%)	28 (73.7%)	22 (88.0%)	104 (89.7%)	338 (87.8%)	<0.001
Disagree	7 (4.7%)	15 (25.9%)	10 (26.3%)	3 (12%)	12 (10.3%)	47 (12.2%)	
Total	148	58	38	25	116	385	

CS/ES/TPD = clinical supervisors, educational supervisors, and training programme directors. PMQ = primary medical qualification.

### Support

Trainees were asked from whom they had received the best support and advice in reflection. The majority stated this came from their supervisors which included clinical supervisors (CS), educational supervisors (ES), and training programme directors (TPD) (58.3%), but high numbers of trainees (26.7%) reported friends and peers as the best source of support (see Table 2). Further analysis of the data revealed significant differences in these responses depending on subregion of the world in which PMQs were achieved (Table 2).

### Is reflection beneficial?

The majority of trainees (69.7%) felt that reflection, as they had experienced it within UK GP training, was beneficial. Differences in responses were seen, depending on the subregion of the world in which PMQs were achieved (Table 2). Trainees who achieved their PMQ in Africa were more likely to report that reflection was beneficial, and those in Europe and Oceania, and the Middle East were less likely to report that reflection was beneficial.

### Training in reflection

**Figure 2.**
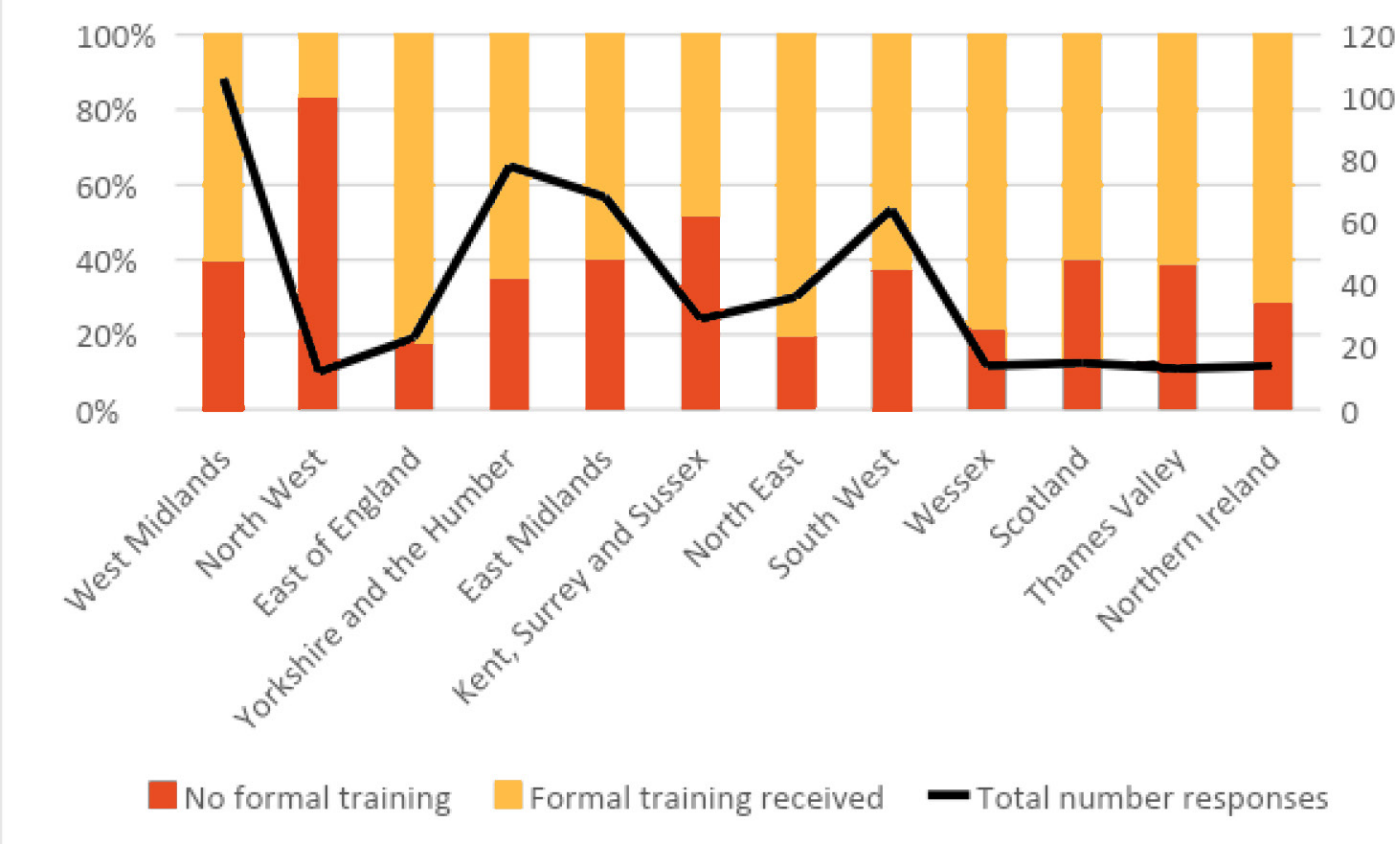
Training in reflection by UK region in which GP training undertaken.

In total, 36.9% of participants reported that they had not had any formal training in reflection. Of those trainees who reported receiving training, this was most likely to be in the form of small group teaching or one-to-one tutorials with supervisors. Looking at whether trainees reported receiving formal training in reflection, differences were seen in response depending on the postgraduate region in which training was being undertaken (*P* = 0.009) (Figure 2).

## Discussion

### Summary

The results of the survey are a valuable addition to the literature in this area and provide new insights about international graduates’ experiences of UK GP training. Despite reporting limited experience of reflection during undergraduate training, the majority of trainees feel that reflection within GP training is beneficial. However, there were significant differences seen in responses to this question depending on the subregion of the world in which PMQs were achieved, with trainees who achieved their PMQ in Africa being much more likely to feel reflection is beneficial than those who completed their PMQ in the Middle East. It is unlikely that this is simply a result of familiarity with reflection, as few trainees from these subregions reported experience of reflection as an undergraduate. Perhaps, then, this arises from much more complex issues of culture and the expectations of education.

Over half of responders viewed supervisors as their best source of support, however those trainees who achieved their PMQ in South Asia were more likely to report that their most valued support came from supervisors than those whose PMQ was achieved in Africa, who were more likely to value support from peers. When one considers that the teaching methods in both these subregions are most likely based on a science-oriented ‘teacher as expert’ model,^6^ such significant differences in response to this question are perhaps surprising. The fact that the difference exists suggests again a level of complexity that necessitates examination in a sociological context.

International graduates have been identified as more vulnerable to the negative consequences of reflection, such as problematic medico-legal complications.^9^ As such, it is concerning to find that high numbers do not report formal training and support to develop their skills in reflection. Trainees reported significant differences in provision of training between regions of the UK. This is unsurprising considering there are currently no national guidelines or protocols for the support of international graduates in UK GP training.

It might be assumed that those regions with higher numbers and proportions of international graduates might be better equipped to support their trainees in developing this key skill for professional development. However, from the results (which must be interpreted with caution considering the low numbers in some regional groups), the region that performed most poorly in reported provision of formal teaching was a Primary Care School where international graduates accounted for 46% of the total number of trainees in the region.

It has been suggested that international graduates are subjected to a ‘cycle of educational deprivation’: they perform less well than their UK colleagues in GP selection centres and are therefore placed in less popular training programmes.^2^ With this in mind, it is disappointing that the study was unable to obtain survey results from London, known to be one of the most popular GP training schemes, which has very low proportions of international graduates, in order to make these comparisons.

### Strengths and limitations

Despite the intention to send the survey to all international graduates undertaking GP training in the UK, the survey was not circulated in 2 of the 14 training regions (despite multiple attempts to contact senior regional training figures) and therefore unintended sample bias may have been introduced.

Non-response bias was minimised by sending at least one reminder to potential participants and by offering an incentive for survey completion. The response rate of 14.2% is much higher than a similar study into the experiences of international graduates,^10^ which reported a 0.7% response rate to electronic survey and 8% to postal survey. The demographic characteristics of the trainees responding to the survey are similar to GMC data in respect to sex, age, and subregion of the world in which PMQs were obtained. Therefore, the data obtained were likely to capture the views of a broadly representative sample of international graduates training in the UK. It could be argued that responders are more likely to have extreme views on reflection, both positive and negative, which may have skewed the results.

Trainees may have felt obliged to agree with statements about the benefit of reflection because this is compulsory to their training, thereby introducing response bias. However, trainees were not afraid to express negative views or concerns about reflection, as is clear from the ongoing analysis of the free text questions; over 80% of responders completed an optional question asking them to describe what they felt was the worst thing about reflection.

It is important to acknowledge that trainees were reporting teaching and support received retrospectively. There is the possibility of recall bias; trainees may have forgotten teaching sessions delivered or that there was lack of attendance and or engagament from trainees with available sessions.

### Comparison with existing literature

The findings presented here confirm that international graduates are unlikely to have experienced reflection as part of their undergraduate training. This has been suggested in previous articles, but not conclusively evidenced.^6^

One of the criticisms of previous research involving international graduates was that interventions were often developed which treat the trainees as a homogenous group,^3^ without accounting for complex sociological differences. Analysis of the data presented here shows significant differences in experience of reflection as an undergraduate, sources of support most highly valued by trainees, and overall views of the benefit of reflection depending on subregion of the world in which PMQs were achieved. This confirms the importance of ‘embracing complexity’ when it comes to developing interventions for this diverse group of trainees.

### Implications for practice

The findings presented here offer new insights into international graduates’ experiences of reflection, confirming some previous assumptions about undergraduate exposure to reflection, but also offering new insights into sources of support, provision of training and perceived benefits of engaging with reflection. The results have also identified significant differences in the reported provision of training, and support across the different regional GP schools; there may therefore be some benefit from developing national guidance or protocols in how to support international graduates in their transition to UK GP training. Any intervention developed must consider the vast diversity in culture and experience within this group of trainees and consider the individual needs of each trainee.
